# Measuring the variability of personality traits with interval responses: Psychometric properties of the dual-range slider response format: Measuring variability with interval responses

**DOI:** 10.3758/s13428-024-02394-4

**Published:** 2024-04-12

**Authors:** Matthias Kloft, Jean-Paul Snijder, Daniel W. Heck

**Affiliations:** 1 Department of Psychology, University of Marburg, Gutenbergstr. 18, 35032 Marburg, Germany; 2 Heidelberg University, Heidelberg, Germany

**Keywords:** Continuous bounded responses, Item response theory, Multitrait-multimethod, Personality, Trait variability

## Abstract

Measuring the variability in persons’ behaviors and experiences using ecological momentary assessment is time-consuming and costly. We investigate whether interval responses provided through a dual-range slider (DRS) response format can be used as a simple and efficient alternative: Respondents indicate variability in their behavior in a retrospective rating by choosing a lower and an upper bound on a continuous, bounded scale. We investigate the psychometric properties of this response format as a prerequisite for further validation. First, we assess the test–retest reliability of factor-score estimates for the width of DRS intervals. Second, we test whether factor-score estimates of the visual analog scale (VAS) and the location of DRS intervals show convergent validity. Third, we investigate whether factor-score estimates for the DRS are uncorrelated between different personality scales. We present a longitudinal multitrait-multimethod study using two personality scales (Extraversion, Conscientiousness) and two response formats (VAS, DRS) at two measurement occasions (6–8 weeks apart) for which we estimate factor-score correlations in a joint item response theory model. The test–retest reliability of the width of DRS intervals was high ($\hat{\rho } \ge .73$). Also, convergent validity between location scores of VAS and DRS was high ($\hat{\rho } \ge .88$). Conversely, discriminant validity of the width of DRS intervals between Extraversion and Conscientiousness was poor ($\hat{\rho } \ge .94$). In conclusion, the DRS seems to be a reliable response format that could be used to measure the central tendency of a trait equivalently to the VAS. However, it might not be well suited for measuring intra-individual variability in personality traits.

## Introduction

One of the prevalent ways of measuring personality traits in psychology is through self-report questionnaires. Established response formats commonly used in these questionnaires require respondents to select one response from a small set of categories (i.e., Likert-type scales; Likert, 1932) or from a continuous range of values (i.e., visual analog scales, VAS; Hayes & Patterson, 1921). In such formats, responding to a statement or question requires that all relevant experiences and behaviors can be captured by a single value. However, indicating a single response value may be a difficult task if the behavior of a respondent varies widely across different situations (Fleeson & Noftle, 2009). For instance, a respondent might be asked to rate how well the adjective “sociable” describes themselves. While a respondent might be less sociable in a specific situation (e.g., on the job), they might be highly sociable in other situations (e.g., spending a lot of time with their family or frequently meeting up with friends), or depending on their mood, they might have different preferences for social interactions even in similar situations. In such cases, a single response option forces respondents to make a compromise between different situations or intensities of experienced behavior.

Similarly to the case of observed items (i.e., statements or questions), standard measurement models such as factor analysis assume that *latent traits* can also be represented by a single, fixed value for each person (i.e., the true score in classical test theory; Lord, Novick, & Birnbaum, 1968). Substantively, this means that each person has a single, true value on the trait, in our example, sociability. Differences between individuals are then described by the variance of the corresponding latent-trait values. The deviation of an observed response from the fixed value on the latent trait is assumed to be due to measurement error only.

In contrast to this conceptualization, whole trait theory (Baird, Le, & Lucas, 2006; Fleeson, 2001; Fleeson & Jayawickreme, 2015) views the latent trait not as a fixed value, but as a distribution of behaviors and states across situations within a person. Each trait distribution is described by two properties, which are assumed to be stable characteristics of a person: first, the central tendency (i.e., what is usually referred to as the trait), and second, the variance of the distribution (i.e., the intra-individual variability of states). This means that a person can show different behaviors across different situations. Throughout a longer period of time, similar situations are likely to reoccur, and the respondent will exhibit a certain amount of cross-situational consistency in their behavior over these similar situations, thus generating a stable person-characteristic distribution of experienced sociability (Fleeson & Noftle, 2009). Therefore, when comparing the distributions of behaviors across two non-overlapping time spans (e.g., two different weeks; Baird et al., 2006) or the distributions of a single time span randomly split into two halves (Fleeson, 2001), the two distributions should be similar within a person with respect to their means and variances. To return to the example above, if we were to repeatedly ask a respondent to rate their own experienced sociability over the past few hours, this would result in a certain variability of responses depending on the situations they had just encountered. Retrospective ratings on established response formats such as Likert-type scales show high correlations with the central tendency of a state distribution ($r = .42$ to $r = .56$; Fleeson & Gallagher, 2009). However, the variability of latent states or behaviors cannot be measured by a single, retrospective response to an item. An accurate description of personality should not ignore intra-individual variability in the measurement process. Therefore, a solution is needed that goes beyond providing a single response to an item.

### Measuring variability in states, behaviors, and traits

To measure both central tendency and variability of a distribution of states within a person, previous approaches have focused on repeatedly measuring behaviors and states in longitudinal designs (Fleeson, 2001; Fleeson & Gallagher, 2009). This method is termed ecological momentary assessment (EMA) or experience-sampling method (ESM) and has been considered the gold standard for measuring intra-individual variability across time (Conner, Tennen, Fleeson, & Barrett, 2009; Csikszentmihalyi & Larson, 1987). However, EMA methods come with the drawback of being very time-consuming and costly, since participants have to be compensated for multiple measurement occasions. For a relatively short time span of 14 days and a standard number of five measurements per day (as in Fleeson, 2001), this would amount to 70 measurement occasions participants need to be compensated for. Additionally, EMA methods come with challenges such as attrition, selective participation, altered reporting, and reactivity of respondents (Klumb, Elfering, & Herre, 2009). Given that EMA may often not be feasible due to a limited research budget, it is important to develop and test alternative methods for measuring intra-individual variability of state distributions.

**Fig. 1:**
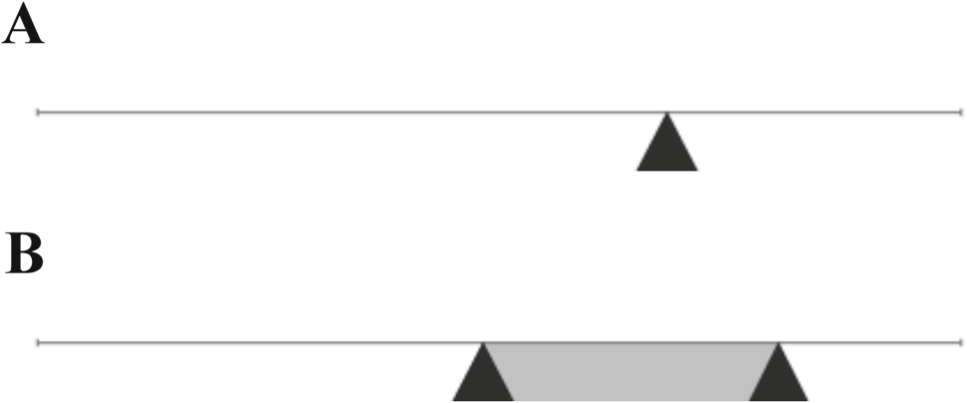
Visual analog scale (**A**) and dual-range slider (**B**). Slider response scales as implemented in the survey software *SoSci Survey* (Leiner, 2019)

To address this gap, we investigate whether a new, more efficient approach can be used that is feasible even in cross-sectional designs. Instead of repeatedly assessing behaviors and states, the central tendency and variability of state distributions may be measured retrospectively by asking respondents to provide interval responses. As shown in Fig. 1B, a dual-range slider (DRS) allows respondents to indicate both a location and variability (via the interval width) for the distribution of states and behaviors experienced in a certain time span. Thus, a person can indicate the variability of an intra-individual state distribution separately for each question or statement.

The DRS response format differs from the more common VAS^1^ (Fig. 1A; Hayes & Patterson, 1921) in that respondents are asked to provide two points rather than a single point on a continuous, bounded response scale. For example, on a *sociability* scale ranging from 0 to 100, a respondent sets both a lower value, such as 40, to indicate that the adjective does not describe them very well in certain situations, and an upper value, such as 90, to indicate that the adjective describes them well in other situations. This corresponds to a response interval with a width of 50 and a location of 65 (computed as the mean of the lower and upper bound). In eliciting retrospective judgments, the instructions should refer to a specific, well-defined context such as a certain reference time period (e.g., the past 12 months) to promote the comparability of ratings across respondents. Note that we do not consider the implementation of elaborated instructions or elicitation methods since the personality items of interest are intended to measure the subjective evaluation of experiences. Accordingly, lower and upper response values are not defined in terms of exact distributional quantiles or other well-defined numeric quantities (e.g., as in probabilistic forecasts of disease incidence and mortality; Taylor & Taylor, 2022; see Discussion). Instead, we consider the DRS to be a straightforward extension of the single-range slider (or VAS) response format. Thus, we used simple and intuitive task instructions (see “Procedure”) to minimize the introduction of construct-irrelevant variance (e.g., stemming from cognitive ability; American Educational Research Association, 2011, Chapter 4).

Whereas the VAS has been established as a reliable and valid response format (Bosch, Revilla, DeCastellarnau, & Weber, 2019; Reips & Funke, 2008), less is known about interval-response formats. Ellerby, Wagner, and Broomell (2022) provided preliminary evidence for the usefulness and validity of interval-response formats by showing that respondents are able to report the variability of a distribution of stimuli via interval responses (i.e., by drawing ellipses on a line segment). The variability of some recurring stimulus or event (e.g., a certain experience across multiple situations) can be measured using two different approaches. Variability can either be assessed directly via an interval response in a retrospective rating of the respective quantity or frequency, or it can be estimated via the aggregation of repeated responses over a given time period (e.g., by assessing the variance of within-person observations in an EMA design). Leertouwer, Schuurman, and Vermunt (2021) found that for a substantial proportion of respondents (50.0% for a positive-affect scale and 60.9% for a negative-affect scale), the retrospective assessment with a single-response format approximated the mean of longitudinal assessments. Fleeson and Gallagher (2009) reported moderate-to-high correlations ($r = .42$ to $r = .56$) between self-report questionnaires and means of EMA measures. Although the concordance between these two data collection methods is not perfect, an interval-response format such as the DRS may save a considerable amount of resources if the intra-individual *variances* of EMA ratings could be similarly approximated by retrospective interval responses.

An ideal approach to investigating the convergent validity of longitudinal single-response measures and retrospective interval-response measures would be to conduct a longitudinal study using the EMA method as a reference for validation (e.g., Leertouwer et al., 2021). However, such an approach comes with substantial costs and challenges, especially when considering the lack of preliminary evidence regarding the validity of the interval-response format for measuring personality. Thus, the present paper uses a weaker, more feasible validation strategy to assess central prerequisites for using interval responses in personality assessment. Specifically, we study the reliability as well as the convergent and discriminant validity of interval-response measures regarding the central tendency and variability of state distributions. For this purpose, we rely on a much simpler longitudinal multitrait-multimethod design (MT-MM; Campbell & Fiske, 1959).

### Research questions

#### RQ1: Test–retest reliability

Whole trait theory posits that the amount of variability of states across situations is a stable characteristic of a person (Fleeson, 2001). We assume that the intra-individual variability of states is reflected in the width of observed interval responses and can be estimated by latent factor scores. If the measurement of intra-individual variability of state distributions is reliable, we should observe a high correlation of the latent factor scores across different measurement occasions (provided the indicated variability is related to a comparable reference time period). A prerequisite for finding such a consistency of individual differences across measurement occasions is a high (test–retest) reliability of the measures obtained through the interval-response format (Anusic, Lucas, & Donnellan, 2012). Hence, if we find that the factor-score estimates of intra-individual variability are highly correlated across measurement occasions, we can conclude that we have (a) high reliability of the interval-response format and (b) a construct that is stable across measurement occasions. In contrast, if factor-score estimates were not highly correlated across measurement occasions, interval responses would not be suitable for measuring any stable personality characteristics.

#### RQ2: Convergent validity of interval locations and single responses

The interval-response format also provides an estimate for the central tendency of a state distribution, namely, the location of a given interval on the response scale (Kloft, Hartmann, Voss, & Heck, 2023). If the response format provides valid measures, the location of an interval response should yield information similar to the slider position in a single-response format of the same type (i.e., a VAS in case of the DRS, see also Fig. 1). Following this logic, ordinary single responses and interval locations, and consequently, the corresponding factor-score estimates representing the central tendency of the latent trait, should be highly correlated within a single measurement occasion. Observing a high correlation between the two types of response formats would indicate convergent validity, meaning that both formats measure the same construct, which is assumed to be the central tendency of a trait. Evidence for the convergent validity of interval-response measures has been found in a previous study by Kloft et al. (2023), and thus, our aim is to replicate this finding.

#### RQ3: Discriminant validity

We expect a high consistency of individual differences across measurement occasions regarding the interval widths and their corresponding factor-score estimates of intra-individual variability. Nevertheless, it is not guaranteed that (a) high consistency is necessarily due to an underlying *uni-dimensional* construct, and (b) the estimates actually reflect the construct we are interested in (i.e., intra-individual variability of a distribution of latent states or behaviors). High consistency could be caused by various mechanisms. In the ideal case, the actual construct we are interested in results in high consistency of intra-individual variability estimates across time. Another possibility is that the responses are influenced by some other construct that we are not interested in. For instance, instead of five personality dimensions (in case of the Big Five), consistency could be caused by a single global trait of intra-individual variability in personality (Baird et al., 2006). Lastly, the most problematic cause of consistency would be a stable preference for certain types of responses (e.g., a general preference for wide or narrow intervals). Such tendencies could be further categorized into specific response styles (for an overview of single-value response styles, see Van Vaerenbergh & Thomas, 2013).

To disentangle different sources of consistency across measurement occasions, one can obtain measures for multiple traits. If interval widths actually measure the intra-individual variability of state distributions, the corresponding factor-score estimates should not be highly correlated across different personality traits (e.g., Extraversion and Conscientiousness) within a single measurement occasion. A low correlation would therefore indicate discriminant validity. Conversely, a high correlation across different traits would indicate that the measures might be influenced by some common mechanism other than the trait-specific variability of respondents’ behaviors.

Our three research questions can be tested in a multitrait-multimethod design with two measurement occasions. Thereby, we provide a first test of the measurement quality of the new interval-response format (i.e., the DRS) in a simple and efficient design. We test our research questions in a longitudinal study using both the single-response format (VAS) and the interval-response format (DRS), as described in detail in the next section.

## Methods

### Study design

**Fig. 2:**
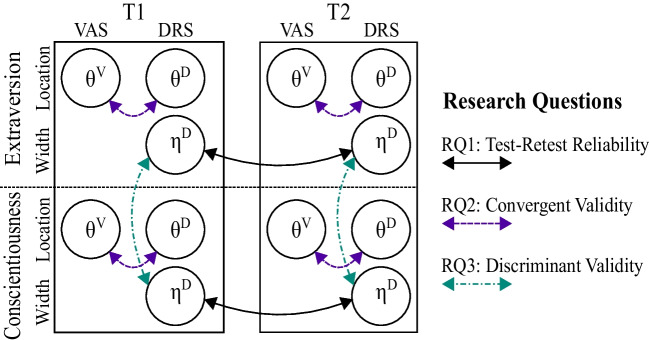
Design of the longitudinal multitrait-multimethod study. VAS = visual analog scale; DRS = dual-range slider; T1 = first measurement occasion; T2 = second measurement occasion, six to eight weeks after first measurement occasion; $\theta$ = person parameter representing the central tendency of an intra-individual state distribution; $\eta$ = person parameter representing the variability of an intra-individual state distribution; RQ1–RQ3 = research questions one through three, arrows indicate correlations that correspond to the respective research question

To assess the psychometric properties of the DRS, we employ a longitudinal multitrait-multimethod (MT-MM) design (Campbell & Fiske, 1959). We collect data for two personality scales (Extraversion, Conscientiousness) that are answered with two different item formats (VAS, DRS) at two measurement occasions with a time lag of 6–8 weeks. Figure 2 gives an overview of the study design. The different types of arrows highlight how our three research questions can be answered by assessing certain correlations between factor scores. First, test–retest reliability (RQ1) corresponds to the correlation of intra-individual variability scores ($\eta ^D$) across the two measurement occasions. Second, convergent validity (RQ2) focuses on the correlation of central-tendency scores ($\theta ^V$ and $\theta ^D$) between the two response formats within each personality scale and measurement occasion. Finally, discriminant validity (RQ3) can be tested based on the correlation of intra-individual variability scores ($\eta ^D$) between the two personality scales and within each measurement occasion. We estimate all factor scores in a joint item response theory (IRT) model that is tailored to continuous, bounded responses. Specifically, we implement the beta response model (BRM; Noel & Dauvier, 2007) for VAS responses and the Dirichlet dual-response model (DDRM; Kloft et al., 2023) for interval responses.

### Sample

We conducted our study at the universities of Marburg and Heidelberg in the summer semester of 2022. All participants were eligible to win one of three vouchers (worth €50 each), while students were also eligible to receive credit toward their study-participation record.

The data set initially included 336 respondents for the first measurement occasion and 244 respondents for the second measurement occasion. We excluded respondents with multiple entries at the first measurement occasion (three respondents, seven entries) or no data at the first measurement occasion (five entries of presumably five respondents, since there were no IDs available for these entries), respondents who indicated at least at one measurement occasion that they did not answer the survey seriously (four respondents, seven entries), respondents who answered the survey questions exceptionally fast^2^ (five respondents, nine entries) or slow^3^ (two respondents, four entries), and outliers regarding means and standard deviations of responses^4^ (11 respondents, 16 entries). In total, we excluded 26 respondents with 43 entries leaving us with a data set of 84 respondents who completed only the first measurement occasion and 224 respondents who completed both measurement occasions. The attrition rate was therefore $27.3\%$. When predicting dropouts by gender, age, educational status, and user device in a logistic regression, none of the predictors were significant at a significance level of $\alpha =5\%$. In our main analyses, we only included those 224 respondents (female: 175, male: 44, diverse: 5) who completed both measurement occasions. The mean age of the final sample was 24.7 years (*SD* $= 7.7$).

### Procedure

For each respondent, the two personality scales (i.e., Extraversion and Conscientiousness) were randomly split into two halves, which in turn were randomly assigned to the two response formats (i.e., VAS and DRS). The items were answered in two blocks which were administered in random order: one block for the VAS and one for the DRS, each containing items from both personality scales. Within each block, the order of items was randomized. Regarding the time lag between the first and the second measurement occasion, we chose a time period of 6–8 weeks, over which personality traits can be assumed to be stable (Anusic et al., 2012). At the second measurement occasion, participants had to complete the same questionnaire again, meaning the assignment of items to response formats was identical to the first measurement occasion, while the order of blocks (VAS, DRS) and items within blocks was again randomized as described above.

Participants had to answer the items in a web browser using visual sliders as shown in Fig. 1. For the VAS format, the instructions asked respondents to indicate how well the presented adjective described their behaviors and attitudes over the past year, more specifically, the past 12 months, using a scale from *not at all* (slider completely to the left) to *fully* (slider completely to the right). For the DRS format, the instructions asked respondents to indicate with a range of values how well the presented adjective described their behaviors and attitudes over the past year, more specifically, the past 12 months, using a scale from *not at all* (slider completely to the left) to *fully* (slider completely to the right). Respondents were instructed that the position of the interval relative to the ends of the scale should indicate how well the adjective described them at an overall level, while the width of the interval should indicate how well the adjective described them across different situations over the last 12 months.

### Measures

#### Extraversion

The Extraversion scale contained 42 person-descriptive adjectives from the *360-PDA* and *525-PDA* inventories of the International Personality Item Pool (IPIP; Goldberg, 1999) in our own German translation. To reach the number of 42 items, we had to extend the original Extraversion scale of the 360-PDA. We did this by computing scale scores for the original Eugene Springfield Community Sample (ESCS) and subsequently selecting adjectives that correlated highly with the original scale scores. We then translated all adjectives to German. Finally, we excluded redundant adjectives as well as adjectives that would be hard to answer using the DRS response format (e.g., more abstract adjectives like “extraordinary”). Four of the translated adjectives represented multiple adjectives from the original inventory (all German adjectives including English translations are provided in the OSF repository: https://osf.io/gfzew/). McDonald’s $\omega _t$ (internal consistency) and McDonald’s $\omega _h$ (*g*-factor saturation) for the original 20-item 360-PDA scale (nine-point Likert-type) in the original ESCS sample were .92 and .63, respectively. McDonald’s $\omega _t$ and McDonald’s $\omega _h$ for the extended 46-item scale (42 translated items plus four redundant items with equivalent German translation) in the original ESCS sample (360-PDA: nine-point Likert-type, 525-PDA: seven-point Likert-type) were .96 and .77, respectively, suggesting the extended scale performed similarly to the original scale.

#### Conscientiousness

The Conscientiousness scale contained 42 person-descriptive adjectives from the 360-PDA and 525-PDA inventories of the IPIP (Goldberg, 1999) in our own German translation. We followed the aforementioned procedure for the extension of the original 360-PDA Conscientiousness scale. McDonald’s $\omega _t$ (internal consistency) and McDonald’s $\omega _h$ (*g*-factor saturation) for the original 360-PDA scale (nine-point Likert-type) in the original ESCS sample were .91 and .68, respectively. McDonald’s $\omega _t$ and McDonald’s $\omega _h$ for our extended 42-item scale in the original ESCS sample (360-PDA: nine-point Likert-type, 525-PDA: seven-point Likert-type) were .94 and .65, respectively, suggesting the extended scale performed similarly to the original scale.

### Item response models for continuous bounded responses

To estimate latent factor scores for Extraversion and Conscientiousness, we fit the BRM (Noel & Dauvier, 2007) to the VAS responses and the DDRM (Kloft et al., 2023) to the DRS responses. Both of these IRT models are tailored for modeling slider responses that are continuous and bounded. Such responses often have skewed distributions due to being bounded by the ends of the response scale (Verkuilen & Smithson, 2012). In the case of interval responses, the model must also account for the dependence of the two response values, which are bounded not only by the ends of the response scale, but also by each other (i.e., the upper bound of the interval response must be above the lower bound). Also, to respond with a more extreme interval location (i.e., move the interval towards one of the response scale’s limits), it is necessary to provide a narrower interval width. This in turn results in a negative non-linear relationship between interval locations and interval widths (for details, see Kloft et al., 2023).

The BRM and DDRM deal with these challenges by considering that continuous responses partition the response scale into two (VAS) or three (DRS) segments that necessarily sum to one. For the VAS, we get a lower (left to the slider) and an upper (right to the slider) component. Analogously, for the DRS, we get a lower component (left to the left slider), a middle component (between the sliders, i.e., the interval width), and an upper component (right to the right slider). These two and three components are then modeled by a beta distribution and a Dirichlet distribution, respectively. To account for person and item differences, these distributions are re-parameterized in terms of person and item parameters, similar to standard IRT models (see Appendices B and C for a detailed definition of both models). Note that we rely on the BRM and the DDRM as measurement models and do not consider these models to provide mechanistic accounts of the underlying response processes (see also “Discussion”).

The IRT modeling approach offers two main advantages for our analyses. First, tailored IRT models account for the interdependencies of the response-scale bounds and responses. This is especially important for the DRS where the lower and upper response values are necessarily dependent. Second, IRT modeling allows us to fit the sparse data that result from randomly assigning half of the items of each personality scale to either response format. Since all respondents answer only half of the items for each response format, half of the data are missing, which can be easily handled in IRT modeling by estimating parameters based on the available responses.

For the latent variables of interest (central tendency and variability of behaviors and states), we estimate the corresponding person parameters (i.e., parameters related to VAS location, DRS location, and DRS width). In Fig. 2, the $\theta ^V$ parameter of the BRM corresponds to the VAS location, whereas the $\theta ^D$ parameter of the DDRM corresponds to the DRS location. These two parameters provide different estimates of the central tendency of the latent-state distribution for a person and correspond to commonly used factor scores for traits such as Extraversion. The third parameter $\eta ^D$ of the DDRM corresponds to the DRS width and aims at capturing the intra-individual variability in the latent-state distribution. All three parameters are estimated for both personality scales and both measurement occasions. Each item loads on only one (or two in case of the DDRM) latent factor(s), and thus, the models assume a simple measurement structure, as in confirmatory factor analysis.

#### Estimation of a joint Bayesian hierarchical model

To investigate our three research questions, we estimate a correlation matrix of person parameters (i.e., factor scores) for different response formats, personality scales, and measurement occasions. More specifically, as shown in Fig. 2, the 12$\times$12 correlation matrix refers to $3 \times 2 \times 2 = 12$ variables, since the location parameter of the BRM (central tendency $\theta ^{V}$) and the two parameters of the DDRM (central tendency $\theta ^{D}$ and variability $\eta ^{D}$) are estimated for both personality traits (Extraversion and Conscientiousness) and both measurement occasions (T1 and T2). Parameter estimation is performed by combining the IRT models for each response format, personality scale, and measurement occasion into a joint Bayesian hierarchical model that assumes a multivariate normal distribution for the 12 person parameters:

1 $$\begin{aligned} (\underbrace{ \theta _{E_1}^{V}, \theta _{E_1}^{D}, \eta _{E_1}^{D}, \,\, \theta _{C_1}^{V}, \theta _{C_1}^{D}, \eta _{C_1}^{D}}_\text {First measurement}, \,\, \underbrace{ \theta _{E_2}^{V}, \theta _{E_2}^{D}, \eta _{E_2}^{D}, \,\, \theta _{C_2}^{V}, \theta _{C_2}^{D}, \eta _{C_2}^{D}}_\text {Second measurement}) \sim \mathcal {MVN} (\varvec{\mu }, \varvec{\Sigma }). \end{aligned}$$

The first six elements of the vector $\varvec{\mu }$ of factor-score means correspond to the first measurement occasion and are set to zero to ensure identifiability of the model. The six remaining means correspond to the second measurement occasion and are freely estimated. The covariance matrix $\varvec{\Sigma }$ can be decomposed into a vector of standard deviations and a matrix of correlations (see Appendix D for mathematical details). Similarly to the means, the six standard deviations for the first measurement occasion are fixed to one to ensure identifiability, whereas the six standard deviations for the second measurement occasion are freely estimated. For all item parameters, we assign weakly informative priors (see Appendix D).

We assume strict measurement invariance across measurement occasions (Moosbrugger & Kelava, 2020, p. 324). Specifically, we estimate only one set of item parameters that is shared across the two measurement occasions. Item parameters include the scaling parameters $\alpha$ (similar to loading parameters in structural equation models), the difficulty parameters $\delta$ (similar to intercept parameters), and the precision parameters $\tau$ (similar to the inverse of residual parameters; for further explanation of the model parameters, see Appendices B and C). We also test the assumption of strict measurement invariance empirically via model comparisons. For this purpose, we fit separate sub-models for each combination of personality scale and response format (see “Measurement invariance”). As a means of comparing models and assessing model fit, we use leave-one-out cross-validation (LOO; Vehtari et al., 2022).

## Results

### Descriptive statistics

Table 1 shows means and standard deviations of responses averaged within each respondent for each personality trait, measurement occasion, and response format. While these averaged responses can be computed directly for the VAS, we use transformed responses for the DRS. Specifically, the mean of the lower and upper bound (i.e., the midpoint between the two) is used to compute the mean scores for the *DRS location*, whereas the difference between the upper and lower bound (i.e., the interval width) is used to compute the mean scores for the *DRS width*. All reverse-coded items have been re-coded for further analyses.

**Table 1:** Descriptive statistics for VAS and DRS responses

		VAS		DRS Location		DRS Width		DRS LB		DRS UB	
Trait	Time	*M*	*SD*	*M*	*SD*	*M*	*SD*	*M*	*SD*	*M*	*SD*
Conscientiousness	T1	66.8	23.4	65.1	21.9	30.1	19.4	50.0	26.8	80.1	20.7
Conscientiousness	T2	66.3	22.1	64.5	21.0	31.2	19.2	48.9	25.7	80.1	20.1
Extraversion	T1	56.6	24.8	54.5	22.5	33.2	21.1	37.9	25.5	71.1	24.2
Extraversion	T2	57.2	23.5	54.4	21.7	34.1	20.6	37.3	24.7	71.5	23.4

*Note*. DRS Location = mean of the dual-range slider’s lower and upper bound; DRS Width = difference of the dual-range slider’s upper and lower bound; DRS LB = dual-range slider lower bound; DRS UB = dual-range slider upper bound

Across the two measurement occasions, the means of the averaged responses were highly comparable. Standard deviations were also very similar, however, there was a trend towards lower variances at the second measurement occasion. A descriptive comparison of the means for the two personality traits indicated higher mean DRS locations for Conscientiousness than for Extraversion. In contrast, mean DRS widths were descriptively larger for Extraversion than for Conscientiousness. This indicates that intra-individual distributions of behaviors and states relevant for Conscientiousness may generally be less variable than those relevant for Extraversion. Alternatively, this result may be due to the dependencies between DRS location and width, meaning that higher responses in DRS location generally go along with lower responses for the DRS width (Kloft et al., 2023). A comparison between VAS and DRS location shows higher means for the VAS. However, we think this comparison should not be interpreted since we chose an arbitrary point inside the interval (i.e., the mean of the two interval bounds) as the DRS location, which might not be the best representation of the DRS to exactly mirror the VAS.

**Fig. 3:**
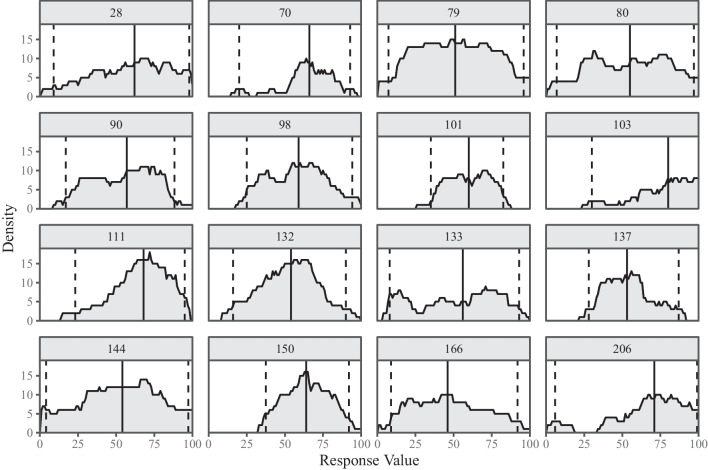
Densities for cumulative intervals for randomly drawn respondents. Solid, vertical line: Median of the respective cumulated interval values. Dashed, vertical lines: 2.5% and 97.5% quantiles of the respective cumulated interval values. Each sub-panel represents all DRS responses of one respondent for the first measurement occasion on the Extraversion personality scale. The plotted cumulative densities are obtained by accumulating all the values contained in the response intervals across items

Figure 3 provides an alternative representation of the data by showing cumulative densities of interval responses for 16 randomly drawn respondents. Each sub-panel represents all DRS responses of one respondent for the first measurement occasion on the Extraversion personality scale. To obtain the cumulative density, all values contained in the response intervals are accumulated across items and the resulting density is plotted (adapted from the interval agreement approach by Wagner, Miller, Garibaldi, Anderson, & Havens, 2015). For instance, for Respondent 144, the density equals $y=12$ for a response value of $x=50$. This means that Respondent 144 included the value 50 in the response intervals for 12 items. Overall, the plotted cumulative densities in Fig. 3 are mostly (approximately) uni-modal or bi-modal. Densities with high mass towards one end of the response scale are mostly skewed towards the middle of the scale.

### Measurement invariance

In the full IRT model fitted in “Model fit, we assume strict measurement invariance across time by constraining all item parameters to be equal across the two measurement occasions (Newsom, 2015, Chapter 2). The assumption of strict measurement invariance is required for meaningful interpretations of differences in factor means and variances across measurement occasions, as well as for correlations of factor scores (e.g., to interpret a correlation between measurement occasions as an estimate of reliability; Moosbrugger & Kelava, 2020, p. 324). Strict measurement invariance requires equal scaling parameters, difficulty parameters, and precision parameters across measurement occasions (note that these parameters have a similar interpretation to loadings, intercepts, and the inverse of residual variances in structural equation models).

To assess different types of measurement invariance, we fitted separate sub-models for each response format and personality scale. We defined four model versions by successively constraining the item parameters for scaling, difficulty, and precision to be equal across measurement occasions. All models were fitted with Stan (Stan Development Team, 2022) in the programming environment R (R Core Team, 2022) via the package *rstan* (Stan Development Team, 2020). For each sub-model, we ran four chains of Stan’s Hamiltonian Monte Carlo (HMC) No-U-Turn Sampler (NUTS; Betancourt, 2018) with 4,000 iterations (plus 4,000 warm-up iterations not considered for analysis) and a thinning factor of 4, resulting in 4,000 posterior samples per parameter. Stan’s adapt_delta parameter was set to .95 for all models (except for three where .99 was used). We checked convergence of MCMC chains by assessing whether the $\hat{R}$ statistic was below 1.05 (Vehtari, Gelman, Simpson, Carpenter, & Bürkner, 2021). We then conducted pairwise comparisons of models via differences in leave-one-out expected log point-wise predictive density (elpd_loo_; Vehtari et al., 2022), starting from the unconstrained baseline models (Tay, Meade, & Cao, 2015). Table 2 presents the results for these model comparisons. The sub-models with the most constraints, assuming strict measurement invariance, demonstrated the best performance in terms of elpd_loo_ across all combinations of personality scales and response formats. This indicates that the predictive power of other model versions did not increase by estimating separate parameters for each measurement occasion. We therefore conclude that the assumption of strict measurement invariance is warranted for all sub-models of the full joint model.

**Table 2:** Measurement invariance model comparisons

	BRM				DDRM			
	Extraversion		Conscientiousness		Extraversion		Conscientiousness	
Model	$\Delta$elpd_loo_	*SE*	$\Delta$elpd_loo_	*SE*	$\Delta$elpd_loo_	*SE*	$\Delta$elpd_loo_	*SE*
Configural	3,436.45	69.45	3,954.91	76.62	10,713.82	95.91	12,060.63	123.94
Metric	49.83	9.13	25.46	7.09	56.15	12.37	16.26	12.34
Scalar	29.33	4.88	29.64	5.95	46.85	8.79	56.00	9.42
Strict	9.07	9.68	29.18	8.84	30.99	7.92	24.63	11.88

*Note*. BRM = beta response model; DDRM = Dirichlet dual response model; $\Delta$elpd_loo_ = leave-one-out expected log point-wise predictive density (first row) and difference in elpd_loo_ compared to the previous row (second to third row), positive values indicate higher elpd_loo_ and consequently better model fit

### Model fit

We fitted the joint Bayesian hierarchical model using the same computing environment and packages mentioned in “Measurement invariance”. We ran four chains of Stan’s HMC NUTS Sampler (Betancourt, 2018) with 6,000 iterations (plus 2,000 warm-up iterations not considered for analysis; see Appendix D for additional information) and a thinning factor of 4, resulting in a total number of 6,000 posterior samples per parameter. Stan’s adapt_delta parameter was set to .95.

We did not observe any pathologies of the MCMC chains as indicated by rstan’s diagnostic summary. MCMC chains converged as indicated by $\hat{R}$ statistics below 1.01 (Vehtari et al., 2021), bulk effective sample sizes of at least 400, and tail effective sample sizes of at least 400. We checked model performance via leave-one-out cross-validation (Vehtari et al., 2022). In a well behaved model, the $\hat{p}_{loo}$ statistic, which is an estimate of the effective number of parameters in a model, should be lower than both the number of observations and the number of parameters. For our model, this was the case, with $\hat{p}_{loo} =$ 2,292 being smaller than the 37,632 observations and 3,548 parameters.

### RQ1: Test–retest reliability

The first research question focuses on the consistency of person parameters across measurement occasions, especially with respect to the parameters that represent the intra-individual variability of the trait ($\eta ^{D}$). Consistency can be assessed through the auto-correlations of parameters across the two measurement occasions shown in Table 3.

**Table 3:** Test–retest reliability: Correlations of person parameters across measurement occasions

Scale	Response format	Parameter	Estimate	95% HDI	$\hat{\rho }< .70$^a^
Extraversion	VAS location	$\theta ^{V}$	$\mathbf {.89}$	[.85, .93]	0.0%
Conscientiousness	VAS location	$\theta ^{V}$	$\mathbf {.90}$	[.86, .94]	0.0%
Extraversion	DRS location	$\theta ^{D}$	$\mathbf {.92}$	[.89, .95]	0.0%
Conscientiousness	DRS location	$\theta ^{D}$	$\mathbf {.87}$	[.83, .92]	0.0%
Extraversion	DRS width	$\eta ^{D}$	$\mathbf {.81}$	[.74, .86]	0.0%
Conscientiousness	DRS width	$\eta ^{D}$	$\mathbf {.73}$	[.65, .81]	22.8%

*Note*. VAS = visual analog scale; DRS = dual-range slider; HDI = highest density interval, a Bayesian credible interval based on the highest posterior density. Estimates are based on the posterior median. Estimates for which the credible interval does not contain zero are printed in mathbf font.

^a^ Percentage of the posterior distribution for the correlation coefficient $\hat{\rho }$ below .70

The auto-correlation was very high for the parameters reflecting the central tendency of a trait (which correspond to the VAS and the DRS location) for both personality scales. These high correlations indicate high test–retest reliability of the corresponding person parameters, which are the factor scores for respondents’ central tendency in Extraversion and Conscientiousness. Meta-analytic estimates for the test–retest reliability of Extraversion and Conscientiousness amount to .85 and .82, respectively (Gnambs, 2014). In comparison, the correlations in our study ranged from .88 to .92 and were thus even above this benchmark. Consequently, we can assume that using the DRS response format will probably have no detrimental effects on the test–retest reliability of factor scores that correspond to the central tendency of a trait.

The temporal consistency of person parameters corresponding to the DRS widths was also satisfactory. Point estimates for both personality scales were above the threshold of .70 (Cicchetti, 1994). However, the estimates had large Bayesian credible intervals, which is probably due to the lower item information of these parameters (i.e., the reduced sensitivity for measuring changes in the latent score; see Kloft et al., 2023). Compared to the aforementioned meta-analytic estimates from prior literature (Gnambs, 2014), these correlations were slightly lower. However, the construct measured by the DRS width might not be perfectly aligned with the central tendency of the trait. Therefore, this comparison is only intended to put the estimated reliability into perspective, not to derive a substantive interpretation. A better reference for comparison may be provided by indicators of stability in EMA studies which use a standard single-response format (Fleeson, 2001; Fleeson & Law, 2015). Indicators of stability are computed as the correlation between the standard deviations of two randomly split halves of the response distribution. Previous studies have found high stability of intra-individual variability with correlations ranging from $r = .55$ to $r = .90$ (mostly $r \approx .80$; five measurements per day for 2–3 weeks; Fleeson, 2001) and from $r = .70$ to $r = .90$ (one measurement for 20 weeks or two measurements for ten weeks in lab sessions by external observers; Fleeson & Law, 2015). As an alternative approach, Baird et al. (2006) investigated stability over time by computing the correlation between the standard deviations of a first and second measurement wave, which were 6–9 months apart. They reported lower stability of intra-individual variability with correlations ranging from $r = .51$ to $r = .66$. These values of previous studies are in a similar range to our model-based estimates of test–retest reliability ($\hat{\rho }=.73$ and $\hat{\rho }=.81$) for a shorter time period of 6–8 weeks.

### RQ2: Convergent validity

The second research question focuses on the convergent validity of VAS and DRS location. Essentially, both response formats are supposed to measure the central tendency of a trait. Convergent validity can be assessed by the correlation of the corresponding person parameters $\theta ^{V}$ and $\theta ^{D}$ shown in Table 4. The estimated correlations were very high (all $\hat{\rho }\ge .88$) for both personality scales and measurement occasions. Hence, we replicated the results of Kloft et al. (2023), who found a correlation of comparable magnitude.

However, it might be possible that the high correlations between the VAS and the DRS response format were merely due to carry-over effects of the instructions. For instance, respondents who completed the VAS block before the DRS block might have been primed to specifically think about a single response value, leading them to subsequently place the lower and upper interval bounds randomly around that value. To test whether the high correlations between VAS and DRS location were due to carry-over effects, we fitted our model separately for the two orders in which the VAS and the DRS blocks were administered. We included only the data from the first measurement occasion. The estimated correlations were highly similar for respondents working on the VAS first and the DRS second (Extraversion: $\hat{\rho } = .90$, 95% HDI [.85, .95]; Conscientiousness: $\hat{\rho } =.91$, 95% HDI [.85, .96]) and for respondents working on the DRS first and the VAS second (Extraversion: $\hat{\rho } = .92$, 95% HDI [.87, .96]; Conscientiousness: $\hat{\rho } = .83$, 95% HDI [.75, .90]). Overall, our results thus present evidence for the assumption that VAS and DRS location provide equivalent measurements of the central tendency of personality traits.

**Table 4:** Convergent validity: correlations of person parameters within personality scales and measurement occasions

Trait	Time	Response formats	Parameters	Estimate	95% HDI
Extraversion	T1	VAS location − DRS location	$\theta ^{V}, \theta ^{D}$	$\mathbf {.93}$	[.90, .96]
Extraversion	T2	VAS location − DRS location	$\theta ^{V}, \theta ^{D}$	$\mathbf {.96}$	[.93, .98]
Conscientiousness	T1	VAS location − DRS location	$\theta ^{V}, \theta ^{D}$	$\mathbf {.88}$	[.84, .92]
Conscientiousness	T2	VAS location − DRS location	$\theta ^{V}, \theta ^{D}$	$\mathbf {.90}$	[.86, .93]
Extraversion	T1	DRS location − DRS width	$$\theta ^{D}, \eta ^{D}$$	.00	$$ [-.13,.12]$$
Extraversion	T2	DRS location − DRS width	$\theta ^{D}, \eta ^{D}$	$-.01$	$[-.13,.12]$
Conscientiousness	T1	DRS location − DRS width	$\theta ^{D}, \eta ^{D}$	$-.11$	$[-.24,.02]$
Conscientiousness	T2	DRS location − DRS width	$\theta ^{D}, \eta ^{D}$	$-.05$	$[-.18,.08]$

*Note*. VAS = visual analog scale; DRS = dual-range slider; HDI = highest density interval, a Bayesian credible interval based on the highest posterior density. Estimates are based on the posterior median. Estimates for which the credible interval does not contain zero are printed in mathbf font

### RQ3: Discriminant validity

The third research question concerns the discriminant validity of the novel part of the DRS response format, the *DRS width* and the corresponding factor scores (i.e., person parameters $\eta ^{D}$ in the model). Evidence for discriminant validity would be indicated by a low correlation of person parameters between different personality scales within each measurement occasion. The relevant correlation estimates are shown in Table 5. The person scores of the DRS width for Extraversion and Conscientiousness were very strongly correlated (all $\hat{\rho }\ge .94$). Substantively, this means that respondents who indicated high intra-individual variability in Extraversion also indicated higher variability in Conscientiousness. This result implies that the discriminant validity of the DRS width for these two personality traits is not satisfactory.

**Table 5:** Discriminant validity: correlations between extraversion and conscientiousness

Response format	Time	Parameter	Estimate	95% HDI
VAS location	T1	$\theta ^{V}$	$\mathbf {.38}$	[.27, .48]
VAS location	T2	$\theta ^{V}$	$\mathbf {.34}$	[.22, .45]
DRS location	T1	$\theta ^{D}$	$\mathbf {.30}$	[.19, .42]
DRS location	T2	$\theta ^{D}$	$\mathbf {.31}$	[.19, .42]
DRS width	T1	$\eta ^{D}$	$\mathbf {.94}$	[.91, .97]
DRS width	T2	$\eta ^{D}$	$\mathbf {.96}$	[.93, .98]

*Note*. VAS = visual analog scale; DRS = dual-range slider; HDI = highest density interval, a Bayesian credible interval based on the highest posterior density. Estimates are based on the posterior median. Estimates for which the credible interval does not contain zero are printed in mathbf font

Again, we compare our correlation estimates against conceptually similar estimates that are obtained in EMA studies. In intensive longitudinal studies, the discriminant validity of intra-individual variability has often been assessed using a multiple regression approach (Baird et al., 2006; Fleeson, 2001; Fleeson & Law, 2015). Essentially, the standard deviation of a response distribution for a certain time period and for a specific trait (e.g., Extraversion) is regressed on the standard deviations of all measured traits (e.g., the Big Five) from a previous time period (Baird et al., 2006). By fitting such a cross-lagged panel model, one can test the unique stability of the intra-individual variability of different traits. Alternatively, if data were collected within a single time period, a regression is fitted for two randomly split halves of the response distribution (Fleeson, 2001; Fleeson & Law, 2015). This corresponds to the correlation between DRS width scores for Extraversion and Conscientiousness in our study, with the difference that we estimated factor scores for intra-individual variability based on retrospective judgments (i.e., DRS width) instead of aggregating responses over multiple repeated measurements. The auto-regressive coefficient in the regression of standard deviations reflects the unique predictiveness of the trait on itself. A large auto-regressive effect signals high discriminant validity since this indicator of unique same-trait stability of intra-individual variability is now statistically controlled for the other traits. Fleeson (2001) and Fleeson and Law (2015) reported high discriminant validity for all Big Five traits with auto-regressive coefficients (unique same-trait stability of intra-individual variability) ranging from .44 to .83 and from .64 to .92, and across-trait regression coefficients (across-trait stability of intra-individual variability) ranging from $-.12$ to .21 and from $-.22$ to .32.

Baird et al. (2006) also used the multiple-regression approach to assess discriminant validity, but mainly focused on increments in the proportion of explained variance $R^2$ (i.e., $\Delta R^2$) as an indicator of unique same-trait stability of the intra-individual variability. In contrast to Fleeson (2001) and Fleeson and Law (2015), Baird and colleagues found only moderate discriminant validity. The amount of variance explained across traits ranged from $R^2 = .26$ to $R^2 = .38$, whereas the amount of additional variance explained by the unique same-trait variability ranged from $\Delta R^2 = .03$ to $\Delta R^2 = .14$. Baird et al. (2006) interpreted these results as evidence for a global trait of intra-individual variability in personality. The high correlation estimates from our model also point in this direction.

**Table 6:** Estimates of the cross-lagged panel model for testing discriminant validity

Parameter	Outcome (T2)	Predictor (T1)	Estimate	95% HDI
$\beta _{11}$	Extraversion	Extraversion	$\mathbf {1.10}$	[0.55, 1.70]
$$\beta _{21}$$		Conscientiousness	$$-0.31$$	$$ [-0.94,0.26]$$
$\beta _{22}$	Conscientiousness	Conscientiousness	0.26	$[-0.42,0.87]$
$\beta _{12}$		Extraversion	0.51	$[-0.10,1.18]$
$\hat{\rho }_{residual}$	−	−	$\mathbf {0.95}$	[0.90, 0.99]
$R^2$	Extraversion	Conscientiousness	0.17	[0.00, 0.46]
$\Delta R^2$		Extraversion	$\mathbf {0.47}$	[0.21, 0.69]
$R^2$	Conscientiousness	Extraversion	0.18	[0.00, 0.46]
$\Delta R^2$		Conscientiousness	$\mathbf {0.36}$	[0.15, 0.59]

*Note.* VAS $=$ visual analog scale; DRS $=$ dual range slider; HDI $=$ highest density interval, a Bayesian credible interval based on the highest posterior density; $\beta$ $=$ auto-regressive and cross-lagged regression coefficients; $\hat{\rho }_{residual}$ $=$ correlation between the residuals of the two factors Extraversion and Conscientiousness at the second measurement occasion. Estimates for which the credible interval does not contain zero are printed in mathbf font

Since our modeling approach only focused on first-order correlations, we also performed a post hoc analysis that more closely mimics the multiple-regression approach by Fleeson (2001), Fleeson and Law (2015), and Baird et al. (2006). On the basis of the estimated correlation matrix of our IRT model, we fitted a cross-lagged panel model that regresses the DRS widths of Extraversion and Conscientiousness at the second measurement occasion on those at the first measurement occasion. We computed one set of regression estimates for each iteration of each MCMC chain. By repeatedly performing this analysis for all MCMC samples of the $4 \times 4$ correlation matrix of all $\eta ^D$ parameters, the results account for estimation uncertainty as reflected by the posterior distribution of our model (for similar approaches see Heck, 2019; Heck, Arnold, & Arnold, 2018). The estimates of the cross-lagged panel model are shown in Table 6.

In contrast to the results of Baird et al. (2006), the incremental variance explained by the same trait was about twice as large as the variance explained across different traits (note, however, that we controlled for only one other trait instead of four). The scores of both traits at the first measurement occasion could explain about two-thirds of the variance of Extraversion at the second measurement occasion and about one-half of the variance of Conscientiousness at the second measurement occasion. The residuals of the DRS width scores for the two personality traits at the second measurement occasion were almost perfectly correlated, as was already the case for the first-order correlations.

Overall, our results provide evidence for a substantial amount of intra-individual variability that is unique to each of the two traits. Also, the regression estimates suggest that the high first-order correlations between the DRS widths of Extraversion and Conscientiousness do not stem from a single underlying construct (e.g., a global, trait-unspecific dimension of intra-individual variability or a general response style). However, we still observed a substantial correlation across traits. The most plausible explanation for the low discriminant validity of DRS width estimates may be that respondents have certain response styles for the use of the DRS response format, which are specific to each measurement occasion.

## Discussion

Our first research question concerned the test–retest reliability of factor scores measuring the central tendency and intra-individual variability of a personality trait (i.e., the person parameters of the model). We found very high test–retest reliability for scores reflecting central tendency and acceptable to high test–retest reliability for factor scores reflecting variability. Our study thus provides evidence that respondents use interval-response formats consistently across different measurement occasions. This is in line with previous research, which found that respondents can adequately express variability using interval responses (Ellerby, Wagner, & Broomell, 2022).

Our second research question concerned the convergent validity of the VAS and the DRS location, both of which are assumed to measure the central tendency of a personality trait (Kloft et al., 2023). We found high correlations between the factor scores reflecting central tendency estimated from VAS and DRS responses, which provides evidence for the convergent validity of the two response formats. Hence, both the single- and the dual-slider response formats can be used interchangeably if one aims at measuring the central tendency of a trait.

The third research question concerned the discriminant validity of the DRS width, which can be assessed by the correlation between factor scores for Extraversion and Conscientiousness. These correlations were estimated to be extremely high, which speaks against the discriminant validity of the DRS width. Our study thus provides evidence that differences in DRS width might not reflect intra-individual variability that is specific to a certain trait. Therefore, interval-response formats might not be suitable for measuring variability in personality traits such as Extraversion.

Our results show high test–retest reliability but insufficient discriminant validity for the DRS width. This raises important questions about the data-generating mechanisms underlying interval responses. Results from previous EMA studies regarding the discriminant validity of personality traits were ambivalent. Fleeson (2001) and Fleeson and Law (2015) reported discriminant validity of intra-individual variability between different traits. In contrast, Baird et al. (2006) found strong correlations between different traits and proposed that a global trait may determine the intra-individual variability of state distributions for all Big Five domains. Our results are more in line with the conclusions of Baird et al. (2006). However, compared to their results, the estimated correlations between Extraversion and Conscientiousness within measurement occasions (see Table 5) were extremely high in our study. Thus, it is unlikely that trait-specific variability or a global trait of variability is the only data-generating mechanism underlying the DRS widths.

Alternatively, it is plausible that respondents are influenced by response styles and may prefer a certain width for their interval responses that is not related to actual variability in a particular trait. In fact, respondents may not be able to retrospectively estimate the amount of intra-individual variability of a specific behavior or state over a certain time period. Instead, they may simply respond with the same, preferred interval width for all items.

To clarify how exactly respondents arrive at the reported intervals, future research could employ cognitive interviews focusing on the underlying response processes (Miller, 2014). Moreover, to increase the validity of response intervals for measuring intra-individual variability, it might be beneficial to rely on more elaborate procedures for eliciting response intervals. For instance, instructions may ask respondents to especially consider *implausible* values when specifying the bounds of the DRS response intervals (i.e., exclusion instead of inclusion instructions; Teigen & Jorgensen, 2005) or to evaluate multiple pre-defined intervals that are later aggregated into a distribution (Haran, Moore, & Morewedge, 2010). However, more evolved elicitation methods or task instructions for interval responses would also be more time-consuming and might reduce the simplicity and the appeal of the DRS format.

As mentioned in the Introduction, more elaborate elicitation methods are often applied in the domain of judgment and decision making when asking respondents to provide uncertainty intervals in forecasting (e.g., Haran et al., 2010; Winman, Hansson, & Juslin, 2004). In such applications, elicitation aims at the proper calibration of reported interval widths in terms of the percentage of intervals that cover a true value or the exact quantiles of a parametric distribution. In contrast, our study focused on the measurement of individual differences in the variability of personality and subjective experiences. In this domain, it is difficult to define numeric target values for a calibration of response intervals. One such target could be certain quantiles of the response distribution of personality items that are repeatedly administered in an EMA study. However, it is unlikely that respondents are motivated and competent to provide retrospective response intervals that correspond to precise numeric quantities (e.g., 20%- and 80%-quantiles). Instead of aiming for such a high and possibly unrealistic standard, the measurement of individual differences in the variability of personality rather focuses on the relative size of response intervals and their rank-ordering between participants.

To better understand the response-generating mechanism, future research should disentangle the influences of a global trait of intra-individual variability, response styles, and unique, trait-specific variability. For instance, one may rely on personality items repeatedly administered in an EMA design as a benchmark for comparison (for directions on how personality items may be implemented in an EMA design, see Andresen, Schuurman, & Hamaker, 2024). In an EMA study, one would expect a high correlation between the intra-personal variances of repeated responses and the widths of retrospective response intervals (see Leertouwer et al., 2021, who used this approach with a single-response format). However, if response styles do have a strong influence on the DRS response format, the question arises as to how much of the test–retest reliability of the DRS width can be attributed to these response styles and how much is due to the actual variability in personality traits. Future research should thus compare the DRS width for different applications. For instance, more objective judgment or forecasting tasks (e.g., interval forecasts for the percentages of parties in election outcomes) might result in improved discriminant validity compared to subjectively anchored personality scales such as those used in the present study.

To the best of our knowledge, our study presents the first empirical assessment of the test–retest reliability and the convergent and discriminant validity of interval responses in the personality domain. A major strength of our study is that we used a fully balanced and randomized design, which is suitable for comparisons at the level of aggregated factor scores. Our experimental design ensured that respondents answered each item only once per measurement occasion, thus avoiding training or order effects. It also allowed us to administer each item with either of the two response formats, thus also avoiding detrimental effects of a fixed assignment of items to a particular response format. The model-based analysis using tailored IRT models is another strength of our approach. By fitting a latent-variable model, we (a) controlled for measurement error in observed interval responses, (b) accounted for the bounded nature of the slider response formats, (c) handled missing responses due to the partially crossed factorial design, and (d) tested the assumption of strict measurement invariance across time points.

According to the Standards for Educational and Psychological Testing, one can rely on different approaches to collect evidence for the validity of the intended use of a test or, in our case, response format (American Educational Research Association, 2011, Chapter 1). A limitation of our study is that we mainly relied on sources of convergent and discriminant validity, which are rooted in construct validity (Campbell & Fiske, 1959). As mentioned above, an alternative validation approach could focus on the comparison of model-based factor scores, which are based on retrospective interval responses, to the actual variability of intra-individual state distributions as measurable in EMA studies. Using EMA data as a gold standard and benchmark for comparisons offers a promising avenue for future research. However, given the lack of discriminant validity of interval responses in our study, researchers should not be too optimistic regarding the convergent validity of EMA-based estimates and the interval-response format. A more direct source of validity evidence lies in the response process itself (Padilla & Benítez, 2014). Our study partially tapped into this source since the convergent validity of VAS and DRS location implies that respondents did not choose the locations of the response intervals randomly or without deliberation. To investigate whether respondents are using the DRS response format as intended, one may conduct cognitive interviews (Miller, 2014) in future research. Another more general limitation of the DRS is that not all adjectives used in personality scales may be appropriate for being answered with an interval-response format.

In the present article, we investigated the psychometric properties of an interval-response format (i.e., the DRS) in the domain of personality measurement. First, test–retest reliability of the location of interval responses was very high, whereas reliability for the width of interval responses was acceptable to high. Second, we replicated the finding that the location of single- and dual-range slider responses can be equivalently used to measure the central tendency of traits. Third, we found evidence against the discriminant validity of interval widths for measuring intra-individual variability in Extraversion and Conscientiousness. Overall, we thus suggest that the DRS response format may not be well suited for measuring variability in personality traits within a person using retrospective self-report questionnaires.

### Open practices statement

All data and analysis scripts for this article are available at the Open Science Framework (OSF): https://osf.io/gfzew/. The study was not preregistered.

## Appendix A Abbreviations and parameter interpretations

- EMA: ecological momentary assessment
- ESM: experience-sampling method
- VAS: visual analog scale
- DRS: dual-range slider
- IRT: item response theory
- BRM: beta response model
- DDRM: Dirichlet dual response model
- MCMC / HMC: Markov Chain Monte Carlo / Hamiltonian Monte Carlo
- NUTS: No-U-Turn Sampler
- HDI: highest density interval (for a given posterior distribution; Bayesian)
- CI: confidence interval (frequentist) / credible interval (Bayesian)
- LOO: leave-one-out cross validation
- elpd_loo_: expected log pointwise predictive density
- $\widehat{R}$: Statistic for the diagnosis of MCMC convergence
- Model Parameters of the BRM and DDRM:
  - $\theta _i$ (BRM, DDRM): person location (i.e., central tendency)
  - $\delta _j$ (BRM, DDRM): item location (i.e., difficulty)
  - $\eta _i$ (DDRM): person expansion (i.e., variability, uncertainty, etc.)
  - $\gamma _j$ (DDRM): item expansion (i.e., strength to elicit wide intervals)
  - $\tau _j$ (BRM, DDRM): item precision (with regard to both interval locations an widths)
  - $\alpha _j (BRM), \alpha _{\lambda j}, \alpha _{\epsilon j}$ (DDRM): item scaling ($\lambda$: location dimension, $\epsilon$: expansion dimension)
  - Parameter superscripts $\theta ^{\varvec{V}}$ and $\theta ^{\varvec{D}}$: Parameter belongs to the VAS/BRM or DRS/DDRM, respectively

## Appendix B The beta response model (BRM)

If we want to fit the beta response model (BRM; Noel & Dauvier, 2007), we need to make sure that a response $X \in (0,1)$. This means that the two components (from lower scale limit to response value, i.e., the slider, and from response value to upper scale limit) must not be zero to ensure the log-likelihood of the beta distribution will not become $-\infty$ (Stan Development Team, 2022; Verkuilen & Smithson, 2012). If the original scale was, e.g., 0 to 100, this can be insured by transforming the original response $X^*$ with $X= \tfrac{X^*+1}{102}$, which implies that the ends of the scale are padded by $\tfrac{1}{102}$.

Let us further consider the response $X_{ij}$ of respondent $i = 1,\dots , I$ (number of respondents) on item $j = 1, \dots , J$ (number of items). This response is modeled by a beta distribution, where each of the two parameters corresponds to one of the components left and right to the slider, respectively:

2 $$\begin{aligned} X_{ij} \sim \text {Beta}(m_{ij},n_{ij}). \end{aligned}$$

The two parameters of the beta distribution are further re-parameterized in terms of latent person and item parameters:

3 $$\begin{aligned} {\begin{matrix} m_{ij} &{}= \exp [ \alpha _{j} (\theta ^{V}_{i} - \delta ^{V}_{j}) + \tau ^{V}_{j}], \\ n_{ij} &{}= \exp [ -\alpha _{j} (\theta ^{V}_{i} - \delta ^{V}_{j}) + \tau ^{V}_{j}]. \end{matrix}} \end{aligned}$$

We use the same parameter names in the BRM and the DDRM for parameters that have an analog meaning (e.g., $\theta _{i}$ or $\delta _{j}$). These parameters are additionally indexed by the superscript *V* for the VAS/BRM (e.g., $\theta ^{V}_{i}$) and *D* for the DRS/DDRM (e.g., $\theta ^{D}_{i}$).

The person parameter $\theta ^{V}_{i}$ represents the trait level and is related to the item location parameter $\delta ^{V}_{j}$ by subtraction. The difference between these two is scaled by the parameter $\pm \alpha _{j}$, which is the equivalent of a factor loading in SEM. The positive sign of $\alpha _{j}$ for $m_{ij}$ and negative sign for $n_{ij}$ moves the expected value of the beta distribution towards one as $\theta ^{V}_{i} - \delta ^{V}_{j}$ becomes more positive and towards zero as $\theta ^{V}_{i} - \delta ^{V}_{j}$ becomes more negative. The precision parameter $\tau ^{V}_j$ is added to both $m_{ij}$ and $n_{ij}$ and thereby decreases the variance of the beta distribution when it is increased.

## Appendix C The Dirichlet dual response model (DDRM)

The DDRM (Kloft et al., 2023) is a straightforward extension of the BRM (Noel & Dauvier, 2007). Similar to the beta unfolding model for continuous bounded responses (Noel, 2014), it uses a Dirichlet distribution, which is a generalization of the beta distribution to more than one dimension, in our case two (DRS location, DRS width). While the BRM concerns one response value (*X*) that partitions a bounded response scale into two components (i.e., a lower and an upper component), the DDRM concerns two response values ($Y_{L}$ and $Y_{U}$) that partition a bounded response scale into three components (i.e., a lower component, a middle component, which is the width of the response interval, and an upper component).

Similar to the BRM, we first have to transform the original response values $Y^*_{L}$ and $Y^*_{U}$ (here on the scale from 0 to 100) to ensure that none of the components can be zero and consequently the log-likelihood of the Dirichlet distribution will not become $-\infty$ (see Stan Development Team, 2022).

By setting $Y_{L} = \tfrac{Y^*_{L} + 1}{103}$ and $Y_{U} = \tfrac{Y^*_{U} + 2}{103}$ we guarantee that $0< Y_{L}< Y_{U} < 1$.

After these transformations, the three components further considered in the model are given by:

4 $$\begin{aligned} \varvec{Y} = \begin{pmatrix} Y_{L} \\ Y_{U} - Y_{L}\\ 1 - Y_{U} \end{pmatrix}. \end{aligned}$$

The response vector of proportions $\varvec{Y}_{ij}$, i.e., respondent *i*’s response on item *j*, is then modeled by a Dirichlet distribution:

5 $$\begin{aligned} \varvec{Y}_{ij} \sim \text {Dir}(o_{ij},p_{ij},q_{ij}). \end{aligned}$$

This Dirichlet distribution is further re-parameterized with latent IRT parameters:

6 $$\begin{aligned} {\begin{matrix} o_{ij} &{}= \exp \bigl [ \alpha _{\lambda j} (\theta ^{D}_{i} - \delta ^{D}_{j}) + \tau ^{D}_{j}\bigr ], \\ p_{ij} &{}= \exp \bigl [ \alpha _{\epsilon j}(\eta _{i} + \gamma _{j}) + \tau ^{D}_{j}\bigr ], \\ q_{ij} &{}= \exp \bigl [ -\alpha _{\lambda j} (\theta ^{D}_{i} - \delta ^{D}_{j}) + \tau ^{D}_{j}\bigr ]. \end{matrix}} \end{aligned}$$

As in the BRM, in the DDRM, we have a location parameter for each person ($\theta ^{D}_{i}$) and each item ($\delta ^{D}_{j}$). The difference between these two parameters determines the expected location of the response interval on the response scale. Additionally, we have an expansion parameter for each person ($\eta _{i}$) and each item ($\gamma _{j}$). The sum of these two determines the expected width of the response interval. Using the sum instead of the difference leads to a more intuitive interpretation of these parameters, i.e., higher values correspond to wider intervals. While the location parameters can be interpreted in standard terms of person trait level and item difficulty, the expansion parameters represent the trait variability of a person (in this study, but can also represent uncertainty, ambiguity or other constructs in different settings) and the mean trait variability of a particular item (or more general, the strength of an item to elicit wide response intervals). Next, the scaling parameters $\alpha _{\lambda j}$ (location dimension) and $\alpha _{\epsilon j}$ (expansion dimension) work analogously to the factor loadings in SEM. Finally, there is the precision parameter $\tau ^{D}_{j}$, which influences the residual variance of both dimensions at the same time. Therefore, a high precision of an item is achieved when the response intervals are homogeneous both with respect to their locations and their widths.

## Appendix D Bayesian parameter estimation

To investigate our research questions, we estimate a correlation matrix of person parameters (i.e., factor scores) for different response formats, personality scales, and measurement occasions. More specifically, as shown in Fig. 2, the 12$\times$12 correlation matrix refers to $3 \times 2 \times 2 = 12$ variables, since the single parameter of the BRM (central tendency $\theta ^{V}$) and the two parameters of the DDRM (central tendency $\theta ^{D}$ and variability $\eta ^{D}$) are estimated for both personality traits (Extraversion and Conscientiousness) and both measurement occasions (T1 and T2). Parameter estimation is performed by combining the IRT models for each response format, personality scale, and measurement occasion into a joint Bayesian hierarchical model that assumes a multivariate normal distribution for the 12 person parameters:

7 $$\begin{aligned} (\underbrace{ \theta _{E_1}^{V}, \theta _{E_1}^{D}, \eta _{E_1}^{D}, \,\, \theta _{C_1}^{V}, \theta _{C_1}^{D}, \eta _{C_1}^{D}}_\text {First measurement}, \,\, \underbrace{ \theta _{E_2}^{V}, \theta _{E_2}^{D}, \eta _{E_2}^{D}, \,\, \theta _{C_2}^{V}, \theta _{C_2}^{D}, \eta _{C_2}^{D}}_\text {Second measurement}) \sim \mathcal {MVN} (\varvec{\mu }, \varvec{\Sigma }). \end{aligned}$$

The first six elements of the vector $\varvec{\mu }$ of factor-score means correspond to the first measurement occasion and are set to zero to ensure identifiability of the model. The six remaining means correspond to the second measurement occasion and are freely estimated. The covariance matrix $\varvec{\Sigma }$ can be decomposed into a vector of standard deviations and a correlation matrix:

8 $$\begin{aligned} \varvec{\Sigma } = \text {diag} (\varvec{\sigma }) \,\varvec{\Omega } \, \text {diag} (\varvec{\sigma }). \end{aligned}$$

The Cholesky factor decomposition of the correlation matrix (Barnard, McCulloch, & Meng, 2000) is used to assume an uninformative LKJ-Cholesky prior (Lewandowski, Kurowicka, & Joe, 2009):

9 $$\begin{aligned} \begin{aligned} \varvec{\Omega }&= \varvec{\Omega }_L \varvec{\Omega }_L^T, \\ \varvec{\Omega }_L&\sim \text {LKJ-Cholesky}(1). \end{aligned} \end{aligned}$$

Similarly to the means, the six standard deviations for the first measurement occasion are fixed to one to ensure identifiability, whereas the six standard deviations for the second measurement occasion are freely estimated with weakly informative priors:

10 $$\begin{aligned} \begin{aligned} \varvec{\mu }_{\theta 2}, \varvec{\mu }_{\eta 2}&\sim t_{3} (0,1), \\ \varvec{\sigma }_{\theta 2}, \varvec{\sigma }_{\eta 2}&\sim t_{3} (1,1) \text { truncated to }(0,\infty ). \\ \end{aligned} \end{aligned}$$

For all item parameters, we also assign weakly informative priors:

11 $$\begin{aligned} \delta _j{} & {} \sim \mathcal {N}(\mu _{\delta },\sigma _{\delta }), \nonumber \\ \gamma _j{} & {} \sim \mathcal {N}(\mu _{\gamma },\sigma _{\gamma }), \nonumber \\ \mu _{\delta }, \mu _{\gamma }{} & {} \sim t_{3}(0,2), \nonumber \\ \sigma _{\delta }, \sigma _{\gamma }{} & {} \sim t_{3} (0,1) \text { truncated to }(0,\infty ), \nonumber \\ \tau _j{} & {} \sim \mathcal {N}(\mu _{\tau },\sigma _{\tau })\text { truncated to }(0,\infty ), \nonumber \\ \alpha _{j}, \alpha _{\lambda j}, \alpha _{\epsilon j}{} & {} \sim \mathcal {N}(\varvec{\mu }_{\alpha },\varvec{\sigma }_{\alpha }) \text { truncated to }(0,\infty ), \nonumber \\ \mu _{\tau }, \varvec{\mu }_{\alpha }{} & {} \sim t_{3} (0,2) \text { truncated to }(0,\infty ), \nonumber \\ \sigma _{\tau }, \varvec{\sigma }_{\alpha }{} & {} \sim t_{3} (0,1) \text { truncated to }(0,\infty ). \end{aligned}$$

Note that the prior for $\tau _j$ is truncated at zero, although negative values for this parameter are technically admissible. However, negative values can lead to multi-modal densities of the Dirichlet distribution and are thus not appropriate for the DDRM model (in contrast to the unfolding model by Noel, 2014).

## Footnotes

1 Some researchers (e.g., Funke, 2016) distinguish between VAS and single-range sliders in that the VAS has no preselected initial value and the selected response value cannot be dragged onto a different point of the response scale, i.e., it is point and click only. Slider scales provide a preselected default value which has to be changed by dragging the slider to the desired response value. In our implementation, the slider can also be placed by clicking on the scale. For the sake of simplicity, and because the term VAS is widely used in the literature, we also use this term, although the format technically is a hybrid between a slider scale and VAS.

2 Mean response times per item of less than 2–3 s for the blocks of VAS and DRS items, respectively.

3 A response time of 318,807 s for the VAS block and 277,191 s for the DRS block.

4 Above or below three standard deviations for mean scores of all responses across both personality scales, separate for each response format, i.e., VAS, DRS lower bound, DRS upper bound, DRS location, and DRS width.
